# Microbial flora and antimicrobial resistance in dental unit waterlines of Chongqing: An observational cross-sectional laboratory study

**DOI:** 10.1097/MD.0000000000049461

**Published:** 2026-07-03

**Authors:** Yiqi Wang, Xuefan Yang, Qiang Wang, Tianzhanhong Shen, Wangcheng Wang, Jingfu Qiu

**Affiliations:** a College of Public Health, Chongqing Medical University, Chongqing, China; b Chongqing Center for Disease Control and Prevention, Chongqing, China; c Wang Qiang Inheritance Studio, Chinese Medicine Hospital of Qijiang District Chongqing, Chongqing, China.

**Keywords:** antibiotic resistance gene, dental unit waterlines, metagenomic sequencing, microbial flora

## Abstract

To identify pathogenic bacteria in dental water systems and assess microbial diversity and resistance genes, we collected water samples from 35 dental facilities in Chongqing, China. Using the VITEK 2 COMPACT system, we identified 26 strains and 13 species of opportunistic pathogens in 23 samples exceeding the standard limits. In addition, metagenomic sequencing was performed to investigate microbial diversity and resistance genes. Among the 170 collected samples, 78.2% qualified, with no significant variation across samples. However, there was a statistically significant difference in qualifying rates between hospitals of different levels (*χ^2^* = 7.696, *P* = .021). Most bacteria (80.8%) were Gram-negative and non-Enterobacteriaceae, with only 1 type belonging to the Enterobacteriaceae family. Notably abundant resistance genes included *bacA, adeC, mexT, mdfA, adeJ, mdtK, emrB,* and *mdtB*, predominantly associated with multidrug resistance (relative abundance: 71.42%). The contamination of dental unit waterlines is a concern that cannot be overlooked.

## 1. Introduction

The dental department is not only a high-risk area for hospital infections but also one of the indispensable departments in medical institutions. With the continuous development of the social economy, people are placing increasing emphasis on oral health and aesthetics, leading to an increasing number of diagnostic and treatment projects being carried out in the dental department.^[1]^ The dental department faces several primary risk factors for hospital infections, including frequent personnel movement, numerous invasive procedures, and challenges in disinfecting specialized equipment. Frequent personnel movement poses a risk because patients in dental outpatient clinics may not only have oral issues but could also be suffering from or carrying other infectious diseases. Numerous invasive procedures, such as cavity preparation, tooth preparation, root canal treatment, and scaling, often involve body fluids and blood, leading to cross-infection.

Recent years have witnessed a surge in patient infections linked to contamination in dental unit waterlines (DUWLs), with viruses like hepatitis B virus and human immunodeficiency virus isolated from dental handpieces.^[2]^ Key contaminants include *Legionella*, *Pseudomonas, nontuberculous mycobacteria, Staphylococcus aureus,* and occasionally *protozoa, fungi,* and *molds*.^[3–8]^ In response, the Chinese Center for Disease Control and Prevention included dental water quality monitoring in the National Hospital Infection Control Program in 2007, setting a limit of 100 CFU/mL.^[9]^ However, from 2007 to 2009, 65.72% of samples exceeded this limit, and recent data (2019) showed that qualification rates for various dental treatment waters remained below 70%, indicating persistent pollution.^[10]^ To address this, several Chinese provinces and cities have issued local standards, with Beijing setting a 100 CFU/mL limit and outlining disinfection measures.^[11]^ International standards vary, with the US CDC and Australian Dental Association recommending ≤500 CFU/mL, the American Dental Association advocating the same, while European Union standards are stricter, at <100 CFU/mL.^[12,13]^

Currently, China lacks a unified national standard for dental diagnostic and treatment water hygiene. Given Chongqing’s significant population flow, a systematic survey of dental departments in local medical institutions is urgently needed. Notably, pathogenic bacteria detection in Chongqing’s oral water remains unexplored, and China has yet to analyze microbial diversity and resistance genes in this context. Therefore, assessing the primary microbial contaminants and their antibiotic resistance in oral water is crucial for developing effective disinfection strategies and preventing hospital infections, with significant implications for hygiene standard formulation.

## 2. Materials and Methods

### 2.1. Materials

The bioMérieux VITEK 2 COMPACT fully automatic microbial analysis system, together with its gram-negative and gram-positive identification cards, a commercially available turbidity meter, tryptic soy agar (TSA), gram-stain kits, and so on.

### 2.2. Sampling objects

There are 35 comprehensive and specialized hospitals in Chongqing, divided into 12 first-level, 13 second-level, and 10 third-level medical facilities. These hospitals include public dentistry departments and use completely random sampling selected based on practicality and voluntary involvement. Water samples gathered include source water for oral departments, water in storage tanks, water from dental unit handpieces, flushing water, 3-way syringe water, and wastewater from dental scalers.

To cover a wide variety of dentistry unit waterlines and better monitor microbial contamination in dental treatment water, the subsequent water samples gathered mainly consist of 3-way syringes and water from dentistry unit handpieces, which possess intricate cavity structures and provide challenges for disinfection.

### 2.3. Quality control

Before sampling, the outlet was cleaned with 75% alcohol, letting the water flow for 30 seconds. Afterward, the water samples were taken by accumulating 50 mL in a sterile test tube and dispatched for analysis within 4 hours. The quality control measure implemented was a strict aseptic technique.

### 2.4. Methods

#### 2.4.1. Bacterial colony total count detection

Homogenize the water sample, extract 1.0 mL, and transfer it onto a sterile Petri dish. Introduce the nutritional agar medium and ensure thorough mixing to completely integrate the sample with the culture medium. Inoculate 2 parallel Petri dishes for each sample.

Except for source water, get an additional 1.0 mL of other water samples for two 10-fold serial dilutions, then inoculate 2 Petri dishes for each dilution. Incubate at 36.6°C for 48 hours and count the colonies.

Statistical analysis: The *χ*^2^ test was used, with a *P* value of <0.05 and a 95% confidence interval (CI) used to define statistical significance.

#### 2.4.2. Bacterial identification test

A sterile pipette extracts 1.0 mL of the material and introduces it into the nutrient broth for an enrichment culture lasting 48 hours. Subsequently, streak to isolate single colonies, select well-developed colonies exhibiting distinct morphologies on the plate, and move them to TSA medium. Isolate the colonies cultivated for 24 hours from the TSA medium, conduct Gram staining, and, based on the stained findings, pick the appropriate VITEK 2 COMPACT bacterial identification card, then use the Merier fully automated microbiological identification system for bacterial identification.

#### 2.4.3. Microbial diversity analysis and resistance gene analysis

**Figure 1.**
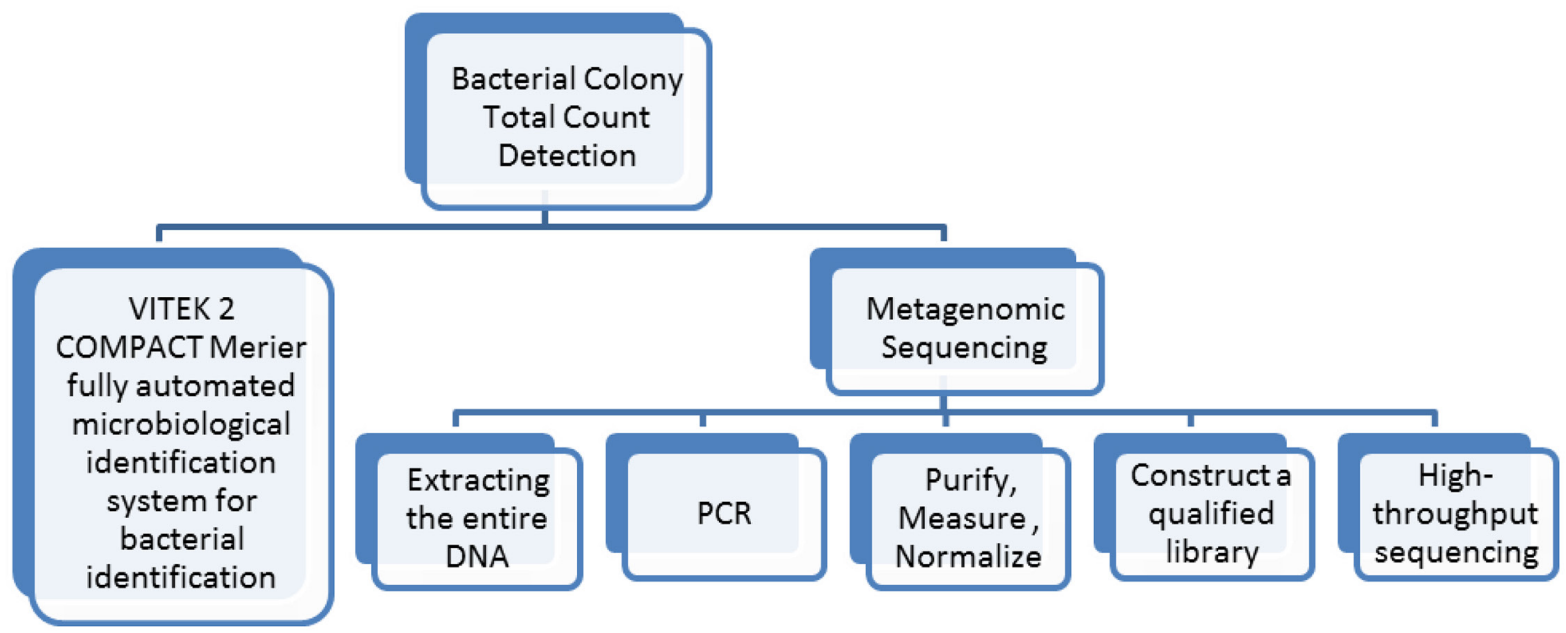
Technical road map. PCR = polymerase chain reaction.

After extracting the entire DNA from the sample, create primers targeting the conserved area, conduct polymerase chain reaction amplification, purify the resultant products, measure and normalize them, and construct a qualified library. The constructed library first underwent a quality control inspection, and libraries that passed quality control were sequenced using the Illumina NovaSeq 6000 platform. Raw image data files obtained from high-throughput sequencing were converted into raw sequencing reads through base-calling analysis. Merge the reads of all samples and assemble them using MEGAHIT, a stitching software based on the De Bruijn graph principle. Based on the overlap relationship between kmers, construct the De Bruijn graph and obtain contigs. Filter Contigs of 800 bp or more for statistical analysis and use them for subsequent analysis. Use SARG 2.0 database software to compare Unigenes with the database (default evaluation ≤ 1^e‐30^). Based on the comparison results of RGI and the abundance information of Unigenes, calculate the relative abundance of each antibiotic resistance gene. The SARG 2.0 version incorporates sequences from the CARD and ARDB databases, as well as the latest sequences from the NCBI-NR database, and was used to analyze the relative abundance of antibiotic resistance genes. For the complete technical roadmap, see Figure 1.

## 3. Results

### 3.1. Basic pollution situation

Since there is no unified standard for oral care treatment water in the country, this water sample standard refers to the “Standards for drinking water quality,” with a limit of 100 CFU/mL being considered acceptable.^[9]^ A total of 170 water samples were obtained from 35 medical institutes, with an overall qualifying rate of 78.2%. The qualifying rate of source water was 100%, whereas the required qualifying rate for other sample water types ranged from around 70% to 80%. Except for source water, there was no statistical difference in the qualification rate of different kinds of oral department diagnostic and treatment water (*χ^2^* = 1.987, *P* = .851, 95% CI: 73.7%–75.6%).

Furthermore, categorized by hospital level, the second-level hospital exhibited the lowest qualification rate at 66.7%, while the qualification rates for the other 2 medical institutions surpassed 80%. A statistically significant difference existed in the qualification rates of oral department diagnostic and treatment services across varying levels of medical institutions (*χ^2^* = 7.696, *P* = .021, 95% CI: 77.8%–80.7%).

### 3.2. Bacterial identification test

Since the qualifying standard for water samples is set at 100 CFU/mL, the colonies formed on the plate after diluting conforming water samples are inadequate. Only water samples that exceed the standards will be tested for microorganisms to reduce possible experimental mistakes. To investigate the source of contamination in the oral department’s diagnostic and treatment water and disinfection protocols, 23 water samples that exceeded the standard were analyzed with the Merier VITEK 2 COMPACT fully automatic microbial identification system, which identified 26 strains and 13 bacterial types.

The detection frequency, ranked from highest to lowest, includes *Sphingomonas paucimobilis* (23.1%), *Ralstonia mannitolilytica* (15.4%), *Granulicatella elegans* (15.4%), *Rhizobium radiobacter* (7.7%), *Cupriavidus pauculus* (7.7%), *Granulicatella adiacens* (3.8%), *Pseudomonas aeruginosa* (3.8%), *Acinetobacter junii* (3.8%), *Acinetobacter baumannii complex* (3.8%), *Sphingobacterium thalpophilum* (3.8%), *Burkholderia cepacia complex* (3.8%), *Brevundimonas diminuta/vesicularis* (3.8%), and *Ralstonia insidiosa* (3.8%), all of which are classified as opportunistic pathogens.

**Figure 2.**
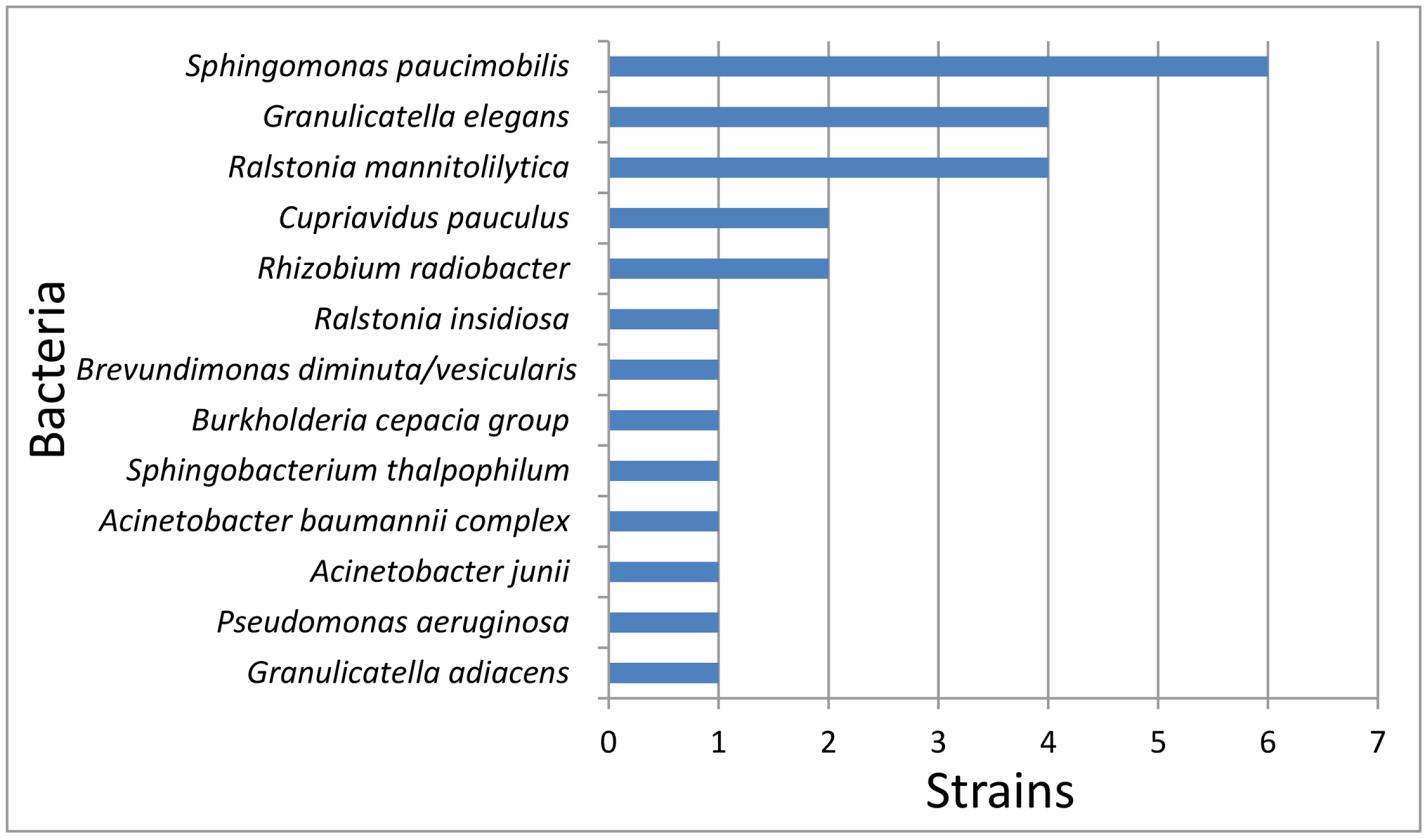
Distribution of bacterial identification plants.

Among them, only 2 (19.2%) species of bacteria, *G. elegans* and *Granulicatella adiacens*, are Gram-positive, while the remaining 11 (80.8%) species are Gram-negative. Among the Gram-negative bacteria, 95.2% are non-Enterobacteriaceae, and only 1 type, *Ralstonia insidiosa*, belongs to the Enterobacteriaceae family. For details, see Table 1 and Figure 2.

**Table 1:** Bacterial identification situation of dental diagnosis and treatment water.

Bacteria	Category	Strains
*Sphingomonas paucimobilis*	Gram-negative, non-Enterobacteriaceae	6
*Ralstonia mannitolilytica*	Gram-negative, non-Enterobacteriaceae	4
*Granulicatella elegans*	Gram-positive	4
*Rhizobium radiobacter*	Gram-negative, non-Enterobacteriaceae	2
*Cupriavidus pauculus*	Gram-negative, non-Enterobacteriaceae	2
*Granulicatella adiacens*	Gram-positive	1
*Pseudomonas aeruginosa*	Gram-negative, non-Enterobacteriaceae	1
*Acinetobacter junii*	Gram-negative, non-Enterobacteriaceae	1
*Acinetobacter baumannii complex*	Gram-negative, non-Enterobacteriaceae	1
*Sphingobacterium thalpophilum*	Gram-negative, non-Enterobacteriaceae	1
*Burkholderia cepacia group*	Gram-negative, non-Enterobacteriaceae	1
*Brevundimonas diminuta/vesicularis*	Gram-negative, non-Enterobacteriaceae	1
*Ralstonia insidiosa*	Gram-negative, Enterobacteriaceae	1

### 3.3. Microbial diversity analysis and resistance gene analysis

To better understand the pathogenic mechanisms of bacteria in dental diagnostic and treatment water, we prefiltered the water samples and used next-generation sequencing and metagenomic sequencing to assess antibiotic resistance. It is well-established in next-generation sequencing practice that raw sequence data must undergo error correction, commonly referred to as “denoising,” to eliminate inaccuracies stemming from various sources such as sequencing chemistry imperfections, polymerase chain reaction amplification biases, and base-calling errors. This denoising process ensures high-quality reads, enhancing the reliability of subsequent analyses.

**Figure 3.**
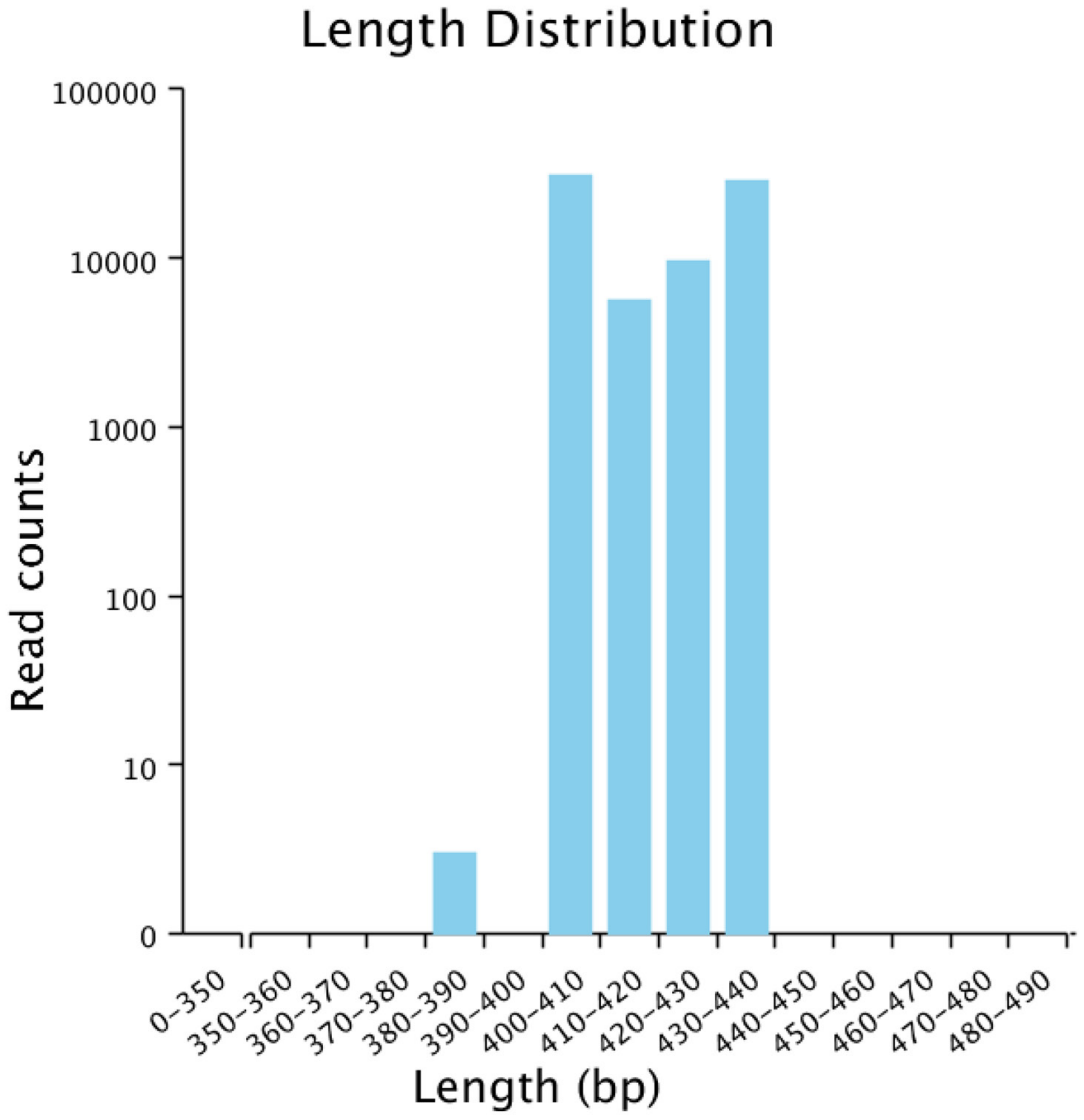
Sequence length distribution map.

The data quality was assessed by measuring characteristics such as the number and length of sequences at each phase. Quality control involved removing reads, including adapters and low-quality sequences, and filtering features with species abundance below 2 in the raw feature table. This step identified and addressed poor-quality bases resulting from sequencing errors. The specific data processing result statistics are presented in Table 2 and Figure 3. The particular number of reads and species at each taxonomic level for the samples can be seen in Table 3. Alpha diversity index statistics refer to the quantitative measures used in ecology to describe species diversity within a particular ecosystem or community. These indices assess a community’s richness (the number of different species) and evenness (the distribution of individuals across those species; Table 4).

**Table 2:** Statistics of sequencing data processing results.

Raw reads	Clean reads	Denoised reads	Merged reads	Nonchimeric reads
88,435	83,081	80,725	77,942	74,579

Raw reads: Raw reads obtained from sequencing. Clean reads: These are the sequences that are free from adapters, have high-quality scores, and do not contain ambiguous bases. Denoised reads: Removing errors or “noise” from the raw sequence reads. Merged reads: The process where overlapping paired-end reads are combined into a single, longer read that represents the entire length of the original DNA fragment based on denoised reads. Nonchimeric reads: Refer to sequences that are not the result of a chimeric event.

**Table 3:** Statistical situation at each taxonomic level.

Statistics types	Kingdom	Phylum	Class	Order	Family	Genus	Species
Reads	74,546	74,130	74,094	69,389	63,951	50,833	9372
Taxonomy levels	2	28	69	159	293	495	543

**Table 4:** Alpha diversity index statistics.

Feature	ACE	Chao1	Simpson	Shannon	PD-whole-tree	Coverage
1051	1051.0	1051.0	0.9765	7.418	80.113	1.0

Feature: The number of OTUs or ASV; ACE: it estimates the total number of species in a community, taking into account the abundance of each species; Chao1: Chao Index, an estimator used to predict the total number of species (OTUs) in a sample; Simpson: Simpson index, this index focuses on the probability that 2 individuals randomly selected from a sample will belong to the same species; Shannon: Shannon–Wiener index, this index measures both richness and evenness by considering the number of species and their relative abundances; PD-whole-tree: a PD metric that quantifies the total branch length of the phylogenetic tree that encompasses all the taxa (OTUs, species, etc.) within a sample or a community. This metric integrates both the species richness and the evolutionary relationships among those species, providing a more comprehensive view of biodiversity than richness or abundance alone; a higher PD-whole-tree value indicates a more significant amount of PD; Coverage: Coverage rate of sample library.

ACE = abundance-based coverage estimator, PD = phylogenetic diversity.

The relative abundance of each resistance gene was determined using the SARG2 database and RGI comparison results, combined with the abundance information of Unigenes. It was found that there is resistance to *bacitracin* (relative abundance: 19.79), *aminoglycoside* (relative abundance: 5.93), *rifamycin* (relative abundance: 4.07), *chloramphenicol* (relative abundance: 1.76), *sulfonamide* (relative abundance: 1.56), *beta-lactam* (relative abundance: 1.21), *tetracycline* (relative abundance: 0.33), *macrolide-lincosamide-streptogramin* (relative abundance: 0.13), with more being multidrug resistance (relative abundance: 71.42). The resistance genes with relatively high abundance mainly include *bacA* (relative abundance: 19.79), *adeC* (relative abundance: 11.45), *mexT* (relative abundance: 10.60), *mdfA* (relative abundance: 10.42), *adeJ* (relative abundance: 9.85), *mdtK* (relative abundance: 8.66), *emrB* (relative abundance: 7.46), *mdtB* (relative abundance: 5.73), and so forth. The specific relative abundances can be seen in Table 5.

**Table 5:** Relative abundance of ARG.

ARG_Category	ARG	Relative abundance	Total
*Aminoglycoside*	*aadA*	1.59	5.93
	*aac3-IA*	0.64	
	*aac2-I*	3.28	
	*bifunctional_aminoglycoside_N-acetyltransferase_and_aminoglycoside_phosphotransferase*	0.05	
	*ant3-Ih-aac6-IId*	0.37	
*Bacitracin*	*bacA*	19.79	19.79
*Beta-lactam*	*metallo-beta-lactamase*	0.82	1.21
	*OXA-21*	0.06	
	*CfxA2*	0.13	
	*TEM-1*	0.05	
	*VEB-3*	0.10	
	*GES-1*	0.05	
*Chloramphenicol*	*cat_chloramphenicol_acetyltransferase*	1.73	1.76
	*cmlA*	0.03	
*Macrolide-lincosamide-streptogramin*	*ermF*	0.06	0.13
	*ermB*	0.07	
*Multidrug*	*emrE*	3.49	71.42
	*mdfA*	10.42	
	*mexF*	1.42	
	*mexT*	10.60	
	*major_facilitator_superfamily_transporter*	1.63	
	*adeC*	11.45	
	*TolC*	0.71	
	*emrB*	7.46	
	*mdtB*	5.73	
	*mdtK*	8.66	
	*adeJ*	9.85	
*Rifamycin*	*ADP-ribosylating_transferase_arr*	4.07	4.07
*Sulfonamide*	*sul1*	0.16	1.56
	*sul4*	1.40	
*Tetracycline*	*tetQ*	0.06	0.33
	*tet39*	0.20	
	*tetA*	0.03	
	*tetM*	0.04	

ARG = antibiotic resistance gene.

## 4. Discussion

The research indicated that the qualification rate for dental diagnostic and treatment water in Chongqing is 78.2%, which is inferior to the qualification rates for routine monitoring parameters in hospitals, including the air in operating rooms, the surfaces of essential medical equipment, the disinfecting agents used, and the hygiene of medical personnel’s hands.^[14–16]^ Therefore, the issue of contamination from dental diagnostic and treatment water requires consideration. All discovered bacteria were opportunistic pathogens, with gram-negative bacteria comprising 80.8%. Among the gram-negative bacteria, almost all were non-Enterobacteriaceae, with just 1 belonging to the Enterobacteriaceae family. This suggests that the contamination of DUWLs mostly originates from prevalent environmental opportunistic infections. However, it does not exclude potential contamination from human microorganisms.

The microorganisms exhibiting elevated detection rates are as follows: *S. paucimobilis* is a gram-negative bacillus susceptible to imipenem and aminoglycosides. This prevalent opportunisticdisease is often found in nature and may facilitate cross-infection inside hospital settings via water supply systems, ventilation systems, and medical apparatus. In recent years, there has been a rising trend in hospital-acquired infections caused by *S. paucimobilis*^[17]^. *R. mannitolilytica* is an opportunistic pathogen capable of infecting individuals with compromised immunity or preexisting conditions, leading to bacteremia, meningitis, respiratory infections, and other diseases. It is most commonly isolated from sputum and can easily cause hospital-acquired infections.^[18,19]^ Its resistance to various antibiotics is variable, making it challenging to choose treatment drugs^[20,21]^. *G. elegans* is a gram-positive coccus widely distributed in nature, existing in environments such as soil, water bodies, and animal intestines. In the medical field, it is also a common pathogen that may cause diseases such as pharyngitis, pneumonia, and sepsis. *Rhizobium radiobacter* is a gram-negative bacterium that can invade the root hairs of leguminous plants, inducing the formation of nodules, usually existing in nodules in various forms (vegetative bacteria), individually or in small groups enclosed in membrane sacs formed by plants, mainly used in the field of water pollution control and ecological restoration. *C. pauculus*, also known as *Ralstonia paucula*, is widely distributed in nature, especially in soil and water bodies. It is a gram-negative, nonfermenting *Bacillus* that can potentially infect patients with weaker immune systems and is an opportunistic pathogen. Broad-spectrum antibiotics such as *Meropenem* are unsuitable for treating *C. pauculus* infections.^[22]^

The prevalence of multidrug resistance is as high as 71.42, and the antibiotic resistance gene with significant abundance is mostly focused in the multidrug resistance category. It can be seen that the expected environmental opportunistic pathogens have become an essential source of contamination threatening the DUWLs. There are also problems with unclear drug resistance and difficulties in choosing treatment drugs. Therefore, it has become a major threat to public health and must not be ignored.

Dental units, classified as class II medical devices in China, are crucial for dental diagnostics and treatments.^[23]^ Their water circuits provide cooling and rinsing functions, supplying water to high-speed turbines and triple syringe guns. However, DUWLs feature narrow pipes prone to contamination and biofilm formation.^[24,25]^ Inadequate disinfection allows microorganisms in the water to contact patients’ oral mucosa, elevating infection risks for immunodeficient patients undergoing oral surgery or with pulp diseases. Furthermore, medical staff’s exposure to aerosols poses significant public health concerns.^[26]^ Thus, oral clinical personnel and infection control staff take water pollution in dental chair units seriously.^[27]^

Moreover, this study has several limitations. For instance, the cross-sectional study design may be subject to seasonal bias, and the study did not directly assess biofilm formation. These aspects require further investigation in future research.

## Author contributions

**Conceptualization:** Yiqi Wang, Jingfu Qiu.

**Data curation:** Yiqi Wang, Xuefan Yang, Wangcheng Wang.

**Formal analysis:** Yiqi Wang, Xuefan Yang, Wangcheng Wang.

**Investigation:** Yiqi Wang, Xuefan Yang, Tianzhanhong Shen, Wangcheng Wang.

**Methodology:** Yiqi Wang, Xuefan Yang, Qiang Wang, Tianzhanhong Shen, Jingfu Qiu.

**Software:** Yiqi Wang.

**Supervision:** Yiqi Wang, Jingfu Qiu.

**Validation:** Yiqi Wang, Jingfu Qiu.

**Project administration:** Xuefan Yang.

**Resources:** Xuefan Yang, Qiang Wang, Tianzhanhong Shen, Jingfu Qiu.

**Funding acquisition:** Jingfu Qiu.

**Visualization:** Jingfu Qiu.

**Writing – original draft:** Yiqi Wang, Xuefan Yang.

**Writing – review and editing:** Jingfu Qiu.
